# Bioaugmentation with *Paenarthrobacter nitroguajacolicus* G1-N1A accelerates atrazine remediation, modulates soil bacterial communities, and alleviates phytotoxicity in common bean

**DOI:** 10.3389/fmicb.2026.1858656

**Published:** 2026-07-22

**Authors:** Jiarui Cui, Haoran Li, Han Gao, Tong Yu, Yongxia Guo, Ming Liu

**Affiliations:** 1 College of Agriculture, Heilongjiang Bayi Agricultural University, Daqing, China; 2 National Coarse Cereals Engineering Research Center, Daqing, China; 3 Key Laboratory of Low-Carbon Green Agriculture in Northeastern China, Ministry of Agriculture and Rural Affairs P. R. China, Daqing, China

**Keywords:** atrazine, bacterial community, bioaugmentation, common bean, G1-N1A, soil enzyme activity

## Abstract

Atrazine, widely applied in maize fields, exhibits strong stability and mobility, leading to soil ecosystem degradation, groundwater pollution, and severe phytotoxicity to succeeding common bean crops in maize-bean rotation systems. Most previously reported atrazine-degrading strains have only been evaluated in liquid culture, while systematic assessments of their efficacy in soil remediation, soil microecology recovery, and crop safety remain limited, restricting their agricultural application. This study aimed to elucidate the atrazine degradation performance of *Paenarthrobacter nitroguajacolicus* G1-N1A in soil, reveal its regulatory effects on soil enzyme activities and bacterial communities, and evaluate its capacity to mitigate atrazine-induced oxidative damage in common bean. Soil incubation degradation experiment, high-throughput sequencing, and pot phytotoxicity experiments were conducted. Atrazine residues in soil were quantified by HPLC, degradation metabolites were identified by LC-MS, the activities of soil sucrase, urease and cellulase were measured using biochemical assay kits, and leaf antioxidant enzyme activities, MDA content, and chlorophyll content were determined in common bean seedlings exposed to a 0-50 mg·kg^−1^ atrazine gradient. The results showed that strain G1-N1A degraded 85.2% of 10 mg·kg^−1^ atrazine within one day in non‑sterile soil, with cyanuric acid identified as the major degradation product via the trzN, atzB, and atzC gene pathways. Inoculation with G1-N1A significantly alleviated the atrazine-induced inhibition of soil sucrase, urease, and cellulase activities, reshaped the bacterial community composition, increased bacterial richness, and accelerated community succession. Pot experiments confirmed that G1-N1A effectively alleviated atrazine phytotoxicity, promoted seedling biomass accumulation, and reduced the elevated activities of antioxidant enzymes as well as MDA accumulation, while maintaining high leaf chlorophyll content across all pollution gradients. Compared with previously reported atrazine-degrading bacteria, strain G1-N1A demonstrates an ultra-fast soil degradation capacity and multi-functional remediation effects, encompassing pollutant removal, soil ecological restoration, and crop protection. This strain represents an environmentally friendly bioaugmentation agent for the green remediation of atrazine-contaminated farmland. This study provides theoretical support and a technical reference for eliminating herbicide residue hazards and ensuring safe production in maize-legume rotation systems in Northeast China.

## 1 Introduction

Atrazine is a triazine herbicide used in corn fields primarily for controlling annual gramineous, broad-leaved, and some perennial weeds (Mac Loughlin et al., 2016). It is commonly applied to the soil surface before and after crop cultivation to prevent the emergence of weeds. China has long been a major producer and user of atrazine, with an annual usage of 1,000 to 1,500 tons (Li et al., 2024). In 2019, the annual consumption of atrazine was nearly 13,000 tons, and the area of land contaminated by atrazine exceeded 1.0 × 10^10^ hectares (Lin et al., 2019). Although usage has gradually decreased in recent years, it is still widely used in corn fields in northern China (Wang and Xie, 2012). When 3.4 kg·ha^−1^ or more atrazine is applied, the subsequent crop yield is significantly reduced or crop failure can occur (Burnside and Moomaw, 1975).

The half-life of atrazine in agricultural soil, which is affected by various complex environmental factors, varies significantly. Due to its high chemical stability and apparent mobility, atrazine is frequently detected in agricultural soils and groundwater (Komtchou et al., 2017). Long-term application leads to gradual accumulation of atrazine in soil, especially in continuous cropping areas, where residual concentrations in soil can sometimes reach the mg·kg^−1^ level. Previous studies have detected atrazine residues as high as 100 mg·kg^−1^ in soil, a value that exceeds the permissible soil limits set by the United States, Canada, the European Union and China (Komtchou et al., 2017), posing a significant phytotoxicity risk to subsequent sensitive crops (Chowdhury et al., 2021). Atrazine has a half-life in soil that can range from 13 to 261 days depending on environmental, and its metabolites can be detected for several years after application (Pan et al., 2023). Atrazine has ecotoxicological effects on biological processes such as soil microbial composition and soil enzyme activities, thereby exerting adverse impacts on soil quality and fertility (Zhang Y. et al., 2023). The results of Zhang Y. et al. (2023) indicate that atrazine and acetochlor exert a synergistic inhibitory effect on soil enzyme activities. High inhibition rates of urease, acid phosphatase, sucrase and catalase were observed under combined atrazine and acetochlor treatment, and the enzyme activities failed to recover to the levels of the herbicide-free group after 28 days. Yang F. et al. (2021) discovered that the application of atrazine had an extremely significant impact on soil cellulase. These residues also impair soil fertility by inhibiting the activities of key enzymes such as urease, sucrase and cellulase, and by disrupting microbial community structure and nutrient cycling (Zhang Y. et al., 2023; Liu et al., 2020; Chen et al., 2014).

In addition to its ecotoxicological effects on fish, terrestrial and aquatic plants, reptiles, and amphibians (Bohn et al., 2011), atrazine poses serious risks to human health. It has been associated with tumors, ovarian, breast, and uterine cancers, as well as leukemia and lymphoma. It has been associated with tumors, ovarian, breast, and uterine cancers, as well as leukemia and lymphoma. As an endocrine disruptor, it interferes with normal hormone function, potentially resulting in congenital disabilities, reproductive tumors, and weight loss in both humans and wildlife (Mukherjee et al., 2019). Consequently, issues related to the removal of atrazine pollution have attracted increasing attention from researchers. Moreover, the soil ecological damage and phytotoxicity to subsequent common bean crops caused by the long-term presence of atrazine residue have become key production problems in the corn–bean rotation system of Northeast China that urgently need to be solved.

Microbial remediation of atrazine-contaminated soil is currently considered an eco-friendly and sustainable “green” remediation method due to its characteristics such as low application cost, environmental safety, and lack of secondary environmental pollution (Wang et al., 2023). In microbial degradation, bacteria, which are characterized by a wide variety of species, ease of cultivation, and strong environmental adaptability, are the main group responsible for degrading atrazine. Microorganisms capable of biodegrading atrazine, including *Pseudomonas* sp. (Mandelbaum et al., 1995), *Citricoccus* sp. (Yang et al., 2018), *Bacillus* sp. (Kolekar et al., 2019), *Agrobacterium* sp. (Struthers et al., 1998), and *Shewanella* sp. (Ye et al., 2016), have been successively isolated and identified. Two typical atrazine metabolic pathways exist among isolated atrazine-degrading bacteria: The first pathway is represented by the classic Gram-negative strain *Pseudomonas* sp. ADP (Esquirol et al., 2018), whose degradation pathway is regulated by six degrading genes, and the complete mineralization pathway of atrazine has been elucidated. Firstly, the strain produces atrazine chlorohydrolase (AC) encoded by the *atz*A gene, which dechlorinates atrazine to form hydroxyatrazine. Secondly, the *atz*B and *atz*C genes of the strain encode hydroxyatrazine ethylaminohydrolase (HAEA) and N-isopropylammelide isopropylaminohydrolase (IAIA) respectively, which catalyze hydrolytic deamination reactions to convert hydroxyatrazine into cyanuric acid. After atrazine is degraded to the intermediate product cyanuric acid, the strain thoroughly degrades atrazine and completes mineralization via cyanuric acid amidohydrolase encoded by the *atz*D gene, biuret hydrolase encoded by the *atz*E gene, and allophanate hydrolase encoded by the *atz*F gene. The second pathway belongs to the Gram-positive strain *Arthrobacter aurescens* TC1 (Strong et al., 2002). The enzyme encoded by the *trz*N gene degrades atrazine into hydroxyatrazine; HAEA encoded by the *atz*B gene further degrades hydroxyatrazine into N-isopropylammelide; ultimately, IAIA encoded by the *atz*C gene converts N-isopropylammelide into cyanuric acid. However, most studies have focused only on degradation efficiency in pure culture, without systematic evaluation of their effects in soil systems and on crop safety. Integrated evidence on whether bioaugmentation-based remediation can simultaneously achieve efficient degradation, soil ecological restoration, and mitigation of crop phytotoxicity is still lacking.

*Paenarthrobacter nitroguajacolicus* G1-N1A is an atrazine-degrading bacterium isolated from the soil of cornfields in Hinggan League, Inner Mongolia Autonomous Region, where there has been long-term atrazine application. The degradation rate of strain G1-N1A on 50 mg·L^−1^ atrazine can reach a maximum of 98.60% within 18 h when the following incubation conditions are used: inoculum size, 1%; temperature, 30 °C; pH, 7.0; and shaker rotation speed, 150 r·min^−1^. The application of herbicides can affect soil microbial abundance, community structure, and enzyme activity (Zhang Y. et al., 2023). Soil enzymes, produced primarily by microorganisms, play vital roles in organic matter decomposition, nutrient cycling, and bioremediation (Chen J. et al., 2021), and their activities can reflect the sensitivity of microorganisms to organic pollutants (Lee et al., 2020). Chen et al. (2014) found that urease activity decreases as the atrazine concentration increases, with fluctuations occurring on the 28th day, whereas sucrase activity is stimulated by low concentrations of atrazine from day 0 to day 7, and catalase activity increases along with the atrazine concentration, with fluctuations appearing on the 7th day. Atrazine residues are known to inhibit urease, sucrase, and cellulase activities (Liu et al., 2020), and to alter the structures and functions of soil microbial communities (Struthers et al., 1998; Strong et al., 2002; Chen J. et al., 2021). Even at low concentrations, atrazine can affect the growth of sensitive crops (Bach et al., 2010; Fang et al., 2015). In the early stage of cultivation, adding *Arthrobacter* strain HB-5 to the soil increases the number of indigenous bacteria, microbial biomass carbon, and the Shannon, Simpson, and Mcintosh diversity indices (Gao et al., 2018). Therefore, changes in microbial community structure can indicate the remediation capability of degrading bacteria and their ecological impacts.

Heilongjiang Province is a major common bean-producing area in China. Common bean is rich in protein and dietary fiber, as well as various vitamins and minerals, and it represents considerable shares in the food processing industry and international market (Los et al., 2018; Kulaz et al., 2022). At present, the over use of atrazine is particularly severe in Heilongjiang Province, located in Northeast China, where rotations of legume crops and corn represent the main farming pattern. However, the over use of atrazine in actual production processes has extremely serious phytotoxic effects on subsequent crops. For example, it reduces the pod setting and germination rates of sensitive crops, like common bean, thereby directly affecting yields. Farming is negatively affected by this phytotoxicity during planting. Thus, there is an urgent need for an efficient, low-risk, and pollution-free alternative to its use in common bean production. It is of great practical significance to develop “green” microbial remediation technologies that can efficiently degrade atrazine, modulated soil health, and protect subsequent common bean crops. Therefore, this study used the highly efficient degrading strain *P. nitroguajacolicus* G1-N1A as the research object and aimed to address this agricultural problem by investigating the strain’s efficient atrazine-degrading characteristics.

This study aimed to determine the degradation ability and metabolic characteristics of the G1-N1A strain on atrazine in soil, analyze its effects on soil sucrase, urease, cellulase activities, and bacterial community structure, and evaluate its regulatory effects on the growth traits, as well as physiological and biochemical indicators, of common bean grown under stress caused by different atrazine concentrations. Although G1-N1A has shown great potential in laboratories, its field performance in real agricultural production environments has not yet been systematically verified. This research gap constitutes a major obstacle for technology to move from theory to practice. This study not only provides an efficient degradation solution for solving the problem of atrazine pollution but also offers theoretical basis and technical support for reducing herbicide residues in crop rotation systems, thereby ensuring the safe production of crops and promoting the further development and application of microbial agents.

## 2 Materials and methods

### 2.1 Chemical reagents and strain

Atrazine (technical grade, purity 97%) was purchased from Yuan Ye Technology Co., Ltd. (Shanghai, China). Soil enzyme kits were purchased from Solarbio Technology Co., Ltd. (Beijing, China). Methanol (chromatographic grade) was used for atrazine extraction and was purchased from Liaoning Quanrui Reagent Co., Ltd. All the other chemical reagents were analytical grade.

The strain *Pseudarthrobacter nitroguajacolicus* G1-N1A (GenBank accession number: OP107895.1) was isolated from the soil of corn fields in Xing’an League, Inner Mongolia Autonomous Region, where atrazine has been applied for a long time. The isolation and degradation verification of this strain were completed in our laboratory.

### 2.2 Soil sample preparation

The soil used in the experiments was farmland soil with no atrazine detected. The physical and chemical properties of the tested soil are as follows: pH 6.82, organic matter 8.4 g·kg^−1^, hydrolyzable nitrogen 74 mg·kg^−1^, available potassium 84 mg·kg^−1^, available phosphorus 10.9 mg·kg^−1^.

It was air-dried, ground and passed through a 2-mm sieve and then divided into two portions. One portion of the soil was sterilized at 121 °C for 30 min, and this process was repeated three times. In total, 300 g of soil was taken for each sample, and 6 mL of a 5 g·L^−1^ atrazine–methanol solution was sprayed evenly onto the soil using a small spray bottle, after which the soil was stirred thoroughly. The mixed soil sample was placed in a fume hood overnight to allow the residual methanol in the contaminated soil to volatilize. At this time, the atrazine content of the soil sample was 100 mg·kg^−1^. Then, 300 g of soil was mixed with 2,700 g of farmland soil free of atrazine, resulting in a 10 mg·kg^−1^ atrazine content in the soil. Finally, 300 g of the contaminated soil was placed into 2-L plastic bottles, ensuring a moisture content of 20%. In addition, soil samples without atrazine were prepared for use as controls.

### 2.3 Strain G1-N1A suspension preparations and soil treatment groupings

Strain G1-N1A was inoculated into LB medium and grown until the OD_600_ reached 1.0. The bacterial cells were collected by centrifugation at 8,000 rpm and washed three times with sterile water. The enriched culture was resuspended in sterile water to obtain a bacterial suspension with a cell density of approximately 1 × 10^8^ CFU·mL^−1^. The bacterial suspension was added to the prepared soil at an inoculation amount of 1% (v·m^−1^). The experiment included the following five treatments: A, unsterilized soil; E, unsterilized soil containing 10 mg·kg-1 atrazine; F, unsterilized soil containing 10 mg·kg^−1^ atrazine + strain G1-N1A; G, sterilized soil containing 10 mg·kg^−1^ atrazine; and H, sterilized soil containing 10 mg·kg^−1^ atrazine + strain G1-N1A. All the treatments were placed in a constant temperature incubator and cultured at 25 °C in the dark. Each treatment had three replicates. Samples were taken on days 1, 3, 5, 7, and 14, and 5 g of soil samples from treatments E, F, G, and H at each timepoint were weighed independently and used for atrazine detection.

Treatment Groups A, E, and F were selected, and samples were taken from each on days 1, 3, and 14. A portion of each sample was used for analyzing soil enzyme activity, and another portion was stored at −80 °C for analyzing atrazine metabolites and microbial communities. Each group of experiments was replicated three times.

### 2.4 Identification and cloning of atrazine degradation-related genes

In this study, *EasyPure*^®^ Genomic DNA kit was used to extract the genomic DNA of strain G1-N1A. After PCR amplification and gel recovery of the atrazine-degrading genes *atz*A, *atz*B, *atz*C, *atz*D, *atz*E, *atz*F (Devers et al., 2004), *trz*N, and *trz*D (Zhang et al., 2019), the products were independently ligated into the pMDTM18-T vector and then transformed into competent *Escherichia coli* DH5α cells. Competent cells were prepared in-house for use in transformation and screening. The PCR products were detected by 1% agarose gel electrophoresis and sent to Jilin Province Kumei Biotechnology Co., Ltd. for sequencing.

### 2.5 Detection and degradation pathway analysis of atrazine

#### 2.5.1 Extraction and determination of atrazine in soil

In total, 5.00 g of the soil sample to be assessed was placed into a 50-mL centrifuge tube, and 15 mL of chromatographic grade methanol was added to the tube. Samples underwent ultrasonication for 2 h, stood at room temperature in the dark for 12 h, underwent ultrasonication again for 1 h, and then were centrifuged at 12,000 rpm at room temperature for 8 min. Each supernatant was passed through a 0.22-μm filter membrane into a brown sample bottle for HPLC detection. The detection conditions were as follows: C18 column (4.6 mm × 250 mm); mobile phase and ratio, methanol:water = 80%:20% (V: V); flow rate, 0.8 mL·m^−1^; column temperature, 40 °C; injection volume, 10 μL, and running time, 9 min. Using the HPLC results, the degradation rate of atrazine was calculated using the following formula:

$$C=\frac{C_{0}\times V}{m}$$

where *C* represents the residual content of atrazine in the sample (mg·kg^−1^), *C*_0_ represents the residual amount of atrazine calculated by the standard curve (mg·L^−1^), V represents the total volume of methanol in the extraction solution, and m represents the mass of the soil sample (g). Information such as standard curve, recovery rate, precision and matrix effect is detailed in Appendix.

#### 2.5.2 Determination of atrazine metabolites in soil

In total, 100 mg of soil was placed into a 2 mL centrifuge tube and 600 μL of 2-chloro-L-phenylalanine (4 mg·L^−1^) prepared in methanol was added. Each sample was then vortexed for 30 s. Afterwards, steel beads were added, samples were ground at 55 Hz for 90 s and sonicated at room temperature for 30 min. Samples were then placed on ice for 30 min and centrifuged at 12,000 rpm at 4 °C for 10 min. Supernatants were passed independently through 0.22 μm membranes. The resulting filtrates were collected in detections vial and used for LC–MS.

The detection conditions were as follows: (1) Thermo Vanquish ultra-high performance liquid system, using ACQUITY UPLC^®^ HSS T3 (2.1 × 100 mm, 1.8 μm) chromatographic column; flow rate, 0.3 mL·min^−1^; column temperature, 40 °C; and injection volume, 2 μL. Scanning was performed using positive and negative ion modes (Zelena et al., 2009). (2) Data was collected using a Thermo Q Exactive mass spectrometer with an electrospray ionization source in both positive and negative ion modes. The spray voltage was set to 3.50 kV for positive ions and −2.50 kV for negative ions. Additionally, the sheath gas was maintained at 40 arb, and the auxiliary gas was set at 10 arbs. The capillary temperature was 325 °C. A full-scan first-order mass spectrometry was performed at a resolution of 70,000 with a scanning range of *m·z^−1^* 100–1,000. High-energy collision dissociation was used for secondary fragmentation, with a collision energy of 30 eV and a secondary resolution of 17,500. The top 10 ions in signal intensity were selected for fragmentation, and dynamic exclusion was employed to remove unnecessary MS/MS information (Want et al., 2013).

### 2.6 Soil enzyme activities and microbial community analysis

#### 2.6.1 Determination of soil enzyme activity levels

Soil sucrase, urease and cellulase were determined using kits from Beijing Solarbio Science & Technology Co., Ltd. and in accordance with the instructions. The standard curve equation for soil sucrase is *y* = 1.68*x* + 0.02 (*R*^2^ = 0.995). The standard curve equation for soil urease is *y* = 0.04*x* + 0.02 (*R*^2^ = 0.955). The standard curve equation for soil cellulase is *y* = 3.66*x* + 0.09 (*R*^2^ = 0.998).

#### 2.6.2 The influence of bacterial strains on the structures of bacterial communities in atrazine-contaminated soil

High-throughput sequencing technology was used to study the structures of soil microbial communities. After extracting genomic DNA from soil samples, the primer pair *B341F* (5’-CCTACGGGNGGCWGCAG-3′) and *B785R* (5’-GACTACHVGGGTATCTAATCC-3′) (Wang et al., 2020) the V3–V4 region of the bacterial 16S rRNA gene and sequencing on the Illumina Novaseq 6000 platform was performed (Hangzhou Kaitai Biotechnology Co., Ltd.). Perform alignment using the SILVA database. The microbial diversity data has been submitted to NCBI with the accession number: PRJNA1473643. Rarefaction curves and other relevant results are provided in the appendix.

After sequencing was completed, the raw off-machine data was assembled, followed by quality control and filtering to obtain valid data for bioinformatics analyses. Vsearch and Cutadapt were used to merge and quality-filter the raw reads. Sequences were clustered with 97% similarity, and sequences with a similarity greater than 97% were grouped into the same Operational Taxonomic Unit (OTU). The generated OTU representative sequences were used for subsequent species annotation. Through an *α*-diversity analysis, Mothur was used to estimate species richness, diversity, and coverage, including Chao and Shannon indices. Weighted UniFrac was used to calculate the distance matrix for a Principal Coordinate Analysis (PCoA).

### 2.7 Pot experiment of common bean and physiological measurements

#### 2.7.1 Effects of G1-N1A-mediated atrazine remediation on fresh and dry weights of common bean seedlings

The strain G1-N1A suspension preparation was as described in Section 2.4. The soil used for the pot experiment was natural non-sterilized farmland soil containing indigenous microbial communities. See Section 2.2 for details on the physical and chemical properties of the soil used.

The experiment comprised the following 10 treatments: CK, 0 mg·kg^−1^ atrazine; CKG, G1-N1A bacterial solution; A, 5 mg·kg^−1^atrazine; AG, 5 mg·kg^−1^atrazine + G1-N1A bacterial solution; B, 10 mg·kg^−1^ atrazine; BG, 10 mg·kg^−1^ atrazine + G1-N1A bacterial solution; C, 20 mg·kg^−1^ atrazine; CG, 20 mg·kg^−1^ atrazine + G1-N1A bacterial solution; D, 50 mg·kg^−1^atrazine; DG, 50 mg·kg^−1^atrazine + G1-N1A bacterial solution.

Each concentration of atrazine in each treatment group was thoroughly mixed with soil separately. Then, 400 grams of the mixed soil from each treatment was placed into square seedling pots with a side length of 9.5 × 9.5 centimeters. Two disinfected common bean seeds were sown in each pot, and each treatment group was equipped with 5 seedling pots, resulting in a total of 10 plants. Seven days after phytotoxicity symptoms appeared, 6 rooted plants were randomly selected from each treatment group, and the soil attached to the roots was rinsed off. The excess water was blotted from the roots with absorbent paper and the fresh weights determined. After killing the greenery by placing in an oven at 105 °C for 30 min, plants were dried at 80 °C until they reached a constant dry weight.

#### 2.7.2 Effects of G1-N1A strain remediation on physiological and biochemical indices of common bean leaves

The preparation of the strain G1-N1A suspension was the same as in Section 2.4, and the planting method and treatments were the same as in Section 2.9.

The superoxide dismutase (SOD), peroxidase (POD), and catalase (CAT) activity levels was determined using the nitrogen blue tetrazolium method (Durak et al., 1993), guaiacol method (Maehly, 1954), and using ultraviolet colorimetry (Maehly, 1954), respectively. The malondialdehyde (MDA) content was determined using the thiobarbituric acid method (Cakmak and Horst, 1991), and the ascorbate peroxidase (APX) activity was determined using the guaiacol method (Zhang B. et al., 2023). The soluble protein content was determined using the Coomassie Brilliant Blue G-250 method (Goicoechea et al., 2000), and chlorophyll was extracted using the alcohol immersion method (Derrien et al., 2017).

### 2.8 Data analyses

One-way analysis of variance (ANOVA) and two-way analysis of variance (ANOVA) was performed using SPSS 25.0 software, and Duncan’s multiple range test was adopted for further comparison of differences between groups. All data are presented as mean ± standard deviation (SD) of three replicate experiments (sample size *n* = 3). A difference was considered statistically significant when *p* < 0.05. The error bars in the figures represent the standard deviation. Graphs were plotted using Origin 2023b and GraphPad Prism software.

## 3 Results

### 3.1 Effects of the G1-N1A strain on atrazine degradation in soil

**Figure 1:**
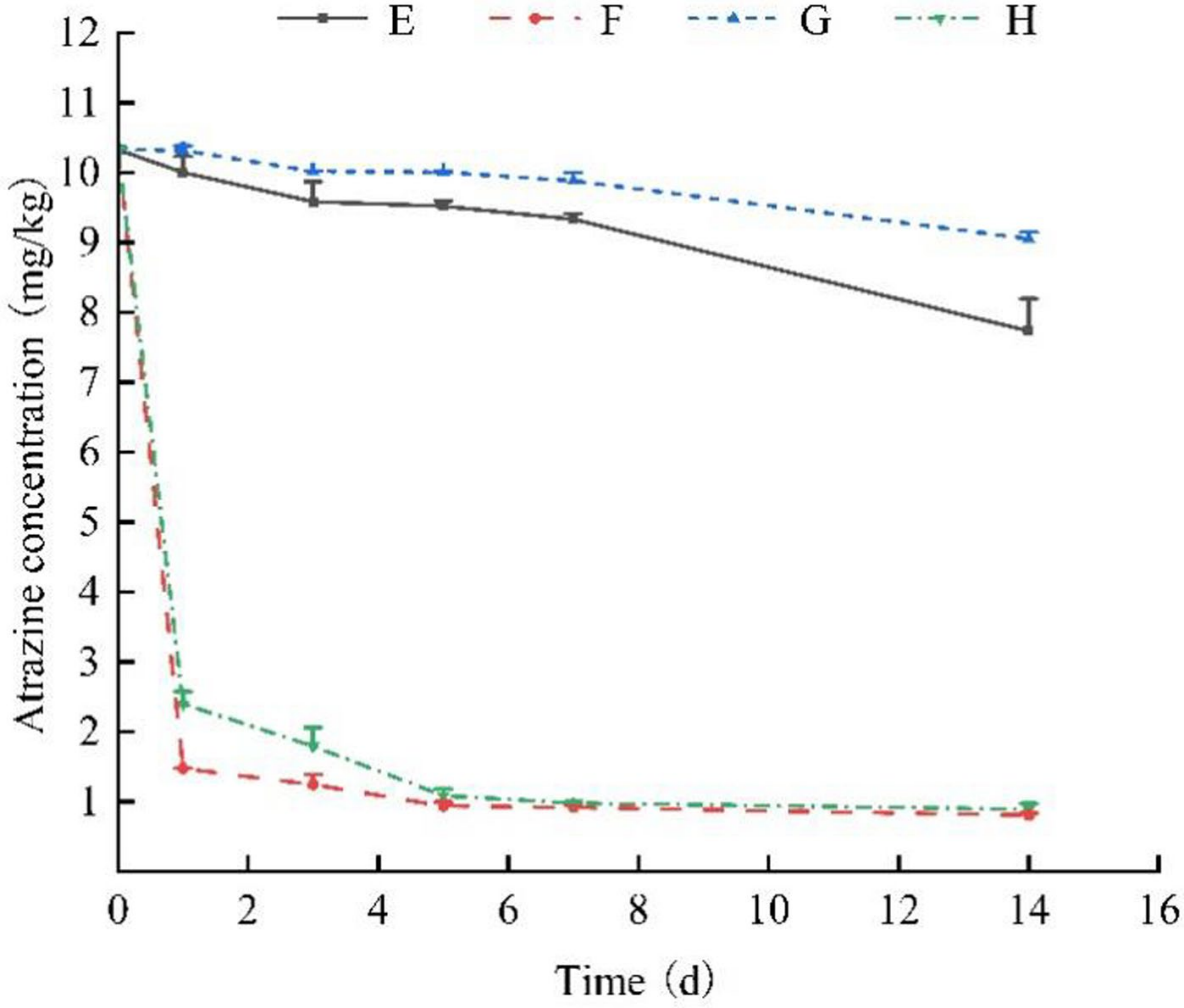
Residual dynamic analysis of atrazine in soil. E: Unsterilized soil + atrazine; F: Unsterilized soil + atrazine + strain G1-N1A; G: Sterilized soil + atrazine; H: Sterilized soil + atrazine + strain G1-N1A.

In non-sterilized soil and sterilized soil supplemented with the G1-N1A strain, the degradation rates of atrazine (10 mg·kg^−1^) within 1 day were 85.20 and 76.00%, respectively. In the non-G1-N1A-supplemented soil, independent of being sterilized, there was almost no atrazine degradation within 1 day (Figure 1). From Days 2 to 5, degradation proceeded slowly, with a higher rate in unsterilized soil than in sterilized soil. After Day 5, the degradation rate reached a maximum of 91%, with no significant difference between the two samples. In non-G1-N1A-supplemented soil, the diversity of the bacterial community structure in the unsterilized soil resulted in a slightly greater atrazine degradation from Days 0 to 3 than in the sterilized soil, but it was not significantly different. From Days 3 to 5, the difference was significant; however, from Days 5 to 14, the difference was no longer significant. Thus, adding strain G1-N1A significantly increased the degradation rate of atrazine in soil.

Two-way analysis of variance (ANOVA) was performed to test the effects of incubation time, soil treatment, and their interaction on atrazine residual concentration. The overall model was highly significant (*F*_23,48_ = 2548.420, *p* < 0.001, *R*^2^ = 0.999). The main effect of incubation time was highly significant (*F*_5,48_ = 2318.311, *p* < 0.001), and the main effect of treatment also showed an extremely significant influence on atrazine residues (*F*_3,48_ = 13002.345, *p* < 0.001). Importantly, a highly significant interaction between incubation time and treatment was detected (*F*_15,48_ = 534.338, *p* < 0.001), indicating that the degradation performance of each treatment varied distinctly across sampling times.

### 3.2 Analysis of strain degradation pathways

#### 3.2.1 Identification and cloning of strain degradation genes

Gene fragments of approximately 500 bp for *atzB*, 600 bp for *atz*C, and 1,380 bp for *trzN* were separately amplified by PCR. No other degradation genes were detected. Sequence alignments of the amplified genes with those in the GenBank database revealed that the *atzB* (OR735533) and *atzC* (OR735534) genes of this strain shared 99 and 100% homology with the *atzB* gene of *Arthrobacter* sp. AK-YN10 (HE716866) and the *atzC* gene of *Arthrobacter* sp. DNS10 (KF453509), respectively. The *trzN* gene (OR735535) exhibited 100% homology with the *trzN* gene of *Arthrobacter* sp. AD30 (FJ161691).

#### 3.2.2 Analysis of atrazine metabolites

To verify the atrazine degradation products resulting from G1-N1A exposure, a blank control without the bacterial treatment was established. LC–MS was used to identify the degradation products. The mass-to-charge ratio (*m·z*^−1^) of the degradation product detected was 128.01. By comparison with known standard compounds and previously reported atrazine metabolites, it is inferred that the final degradation product is cyanuric acid. Based on the degradation genes contained within the strain, the metabolic pathway is preliminarily hypothesized to be as follows: the enzyme encoded by the *trzN* gene degrades atrazine and converts it into hydroxyatrazine; the HAEA encoded by the *atzB* gene then further degrades the hydroxyatrazine into *N*-isopropyl cyanuric acid amide; finally, the IAIA encoded by the *atzC* gene converts the N-isopropyl cyanuric acid amide into cyanuric acid.

**Figure 2:**
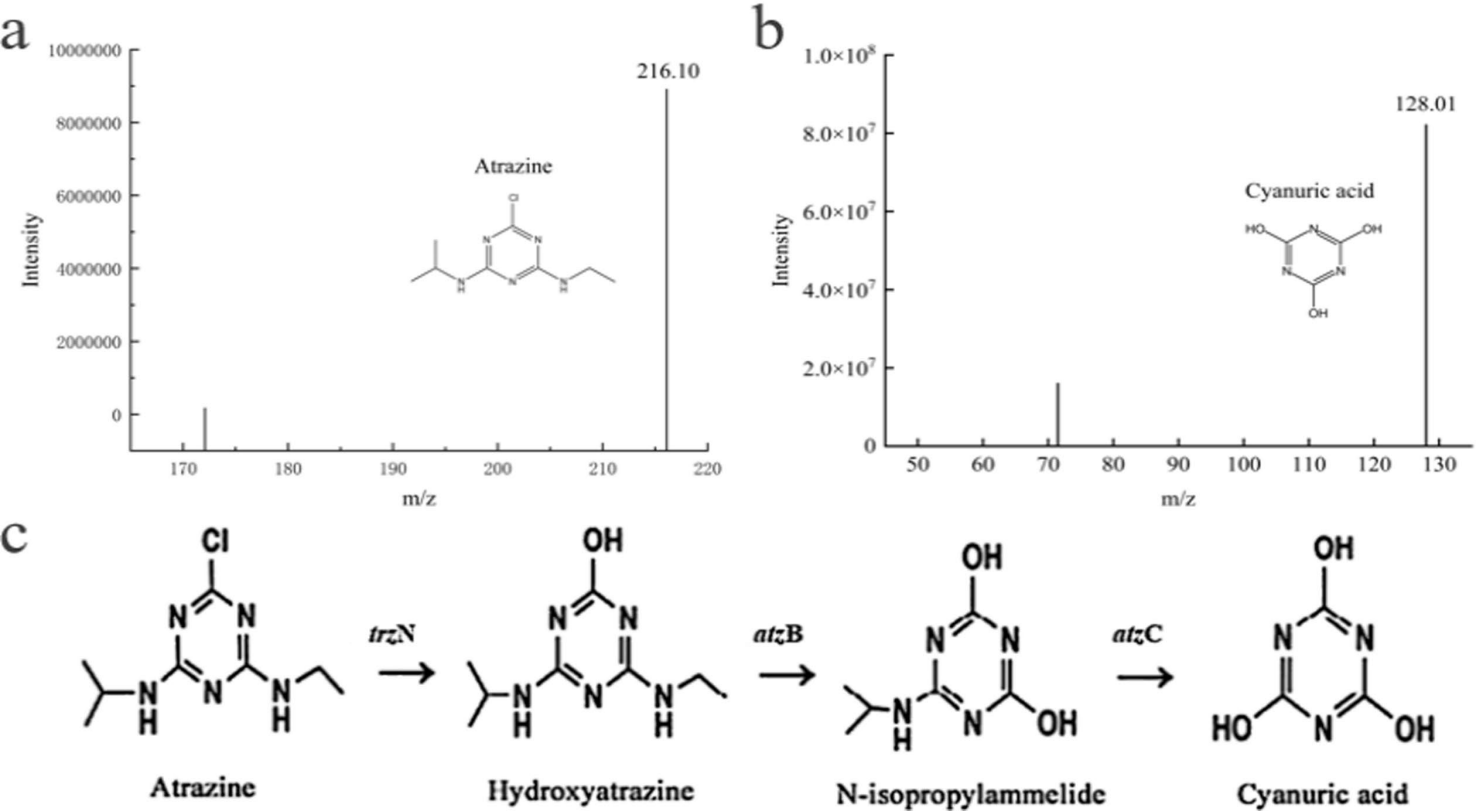
Mass spectra of atrazine **(a)** and its final degradation products **(b)**, as well as the proposed atrazine degradation pathway of strain G1-N1A **(c)**.

Differential metabolites were screened using untargeted metabolomics. Based on the analysis of degradation genes and related degradation products contained in the strain, combined with the complete metabolic pathway of atrazine in the KEGG pathway database,^1^ the degradation pathway of atrazine by G1-N1A was preliminarily speculated (Figure 2).

### 3.3 Effects of atrazine and degrading strains on soil enzyme activities

**Figure 3:**
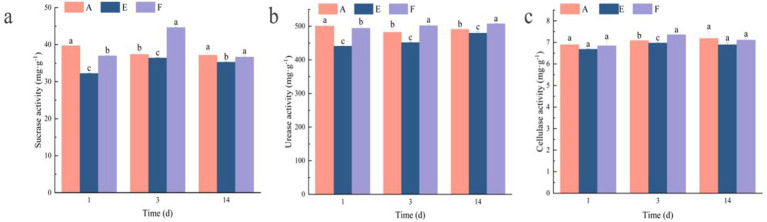
Effects of atrazine and the G1-N1A strain on soil enzyme activities. **(a–c)** Effects of G1-N1A on sucrase **(a)**, urease **(b)**, and cellulase **(c)** activities in soil contaminated by atrazine. The lowercase letters indicate significant difference between different treatments at the same time (*p* < 0.05) as assessed by Duncan’s new complex range method.

As shown in Figure 3a, the soil sucrase activity of Group A (CK) was the highest on Day 1 and then decreased less obviously, from 39.7 mg·g^−1^ to 37.15 mg·g^−1^ by Day 14. In Group E (unsterilized soil + atrazine), sucrase activity in the soil was initially inhibited on Day 1 and then increased. With increased incubation time, there was no significant difference in the sucrase activity among Groups E, A (unsterilized soil), and F. Group F had a higher soil sucrase activity than Group E from Days 1 to 14. Two-way ANOVA was used to analyze the effects of incubation time, soil treatment and their interaction on soil sucrase activity. The overall model was highly significant (*F*_8,18_ = 225.275), (*p* < 0.001), (*R*^2^ = 0.990). The main effect of incubation time exerted an extremely significant influence on sucrase activity (*F*_2,18_ = 196.046), (*p* < 0.001). The main effect of soil treatment was also highly significant, with a larger *F*-value indicating a stronger regulatory effect on sucrase (*F*_2,18_ = 363.516), (*p* < 0.001). A highly significant interaction between incubation time and soil treatment was detected (*F*_4,18_ = 170.768), (*p* < 0.001), which indicated that the temporal variation trend of sucrase activity differed greatly among treatments.

As shown in Figure 3b, the urease activity of control Group A fluctuated within 482–500.67 μg·g^−1^. The soil urease activity in Group E (unsterilized soil + AT) decreased on Day 1. Thus, the presence of atrazine inhibited the soil urease activity, demonstrating an inhibitory effect. From Day 3 to 14, the soil urease activity in Group F (unsterilized soil + AT + strains) increased significantly, demonstrating an activating effect. The soil treatment and their interaction on soil urease activity. The overall fitted model was highly significant (*F*_8,18_ = 104.238), (*p* < 0.001), (*R*^2^ = 0.979). The main effect of incubation time exerted an extremely significant influence on urease activity (*F*_2,18_ = 38.818), (*p* < 0.001). The main effect of soil treatment also showed an extremely significant regulatory effect, with a much larger *F*-value indicating that treatment was the dominant factor altering urease activity (*F*_2,18_ = 312.625), (*p* < 0.001). A highly significant interaction between incubation time and soil treatment was observed (*F*_4,18_ = 32.754), (*p* < 0.001), revealing that the temporal change patterns of urease activity differed markedly among treatments.

As shown in Figure 3c, the presence of atrazine did not significantly affect soil cellulase activity during the early stage of cultivation, while the addition of strain G1-N1A increased soil cellulase activity. The soil cellulase activity of control Group A on Day 1 was 6.89 mg·g^−1^, whereas those of Group E (unsterilized soil + AT) and Group F (unsterilized soil + AT + strain) were 6.68 mg·g^−1^ and 6.84 mg·g^−1^, respectively. These levels were not significantly different. During the first 3 days, significant differences in cellulase activity among the three treatment groups occurred, with the F group having the highest cellulase activity. By 14 days, there were no significant differences in cellulase activity levels among the three treatment groups. Two-way ANOVA was applied to analyze the effects of incubation time, soil treatment and their interaction on soil cellulase activity. The overall model was significant (F_8,18_ = 6.250), (*p* < 0.01), (*R*^2^ = 0.735). The main effect of incubation time exhibited an extremely significant influence on cellulase activity (*F*_2,18_ = 13.729), (*p* < 0.001). The main effect of soil treatment was also statistically significant (*F*_2,18_ = 8.270), (*p* < 0.01), and the *F*-value indicated that incubation time contributed more to the variation of cellulase activity than soil treatment. Notably, the interaction between incubation time and soil treatment was not significant (*F*_4,18_ = 1.500), (*p* = 0.244), which suggested that the temporal variation trends of cellulase activity were consistent across all treatments.

### 3.4 Effects of atrazine and degrading strains on soil microbial communities

#### 3.4.1 The abundance levels of soil microorganisms

**Figure 4:**
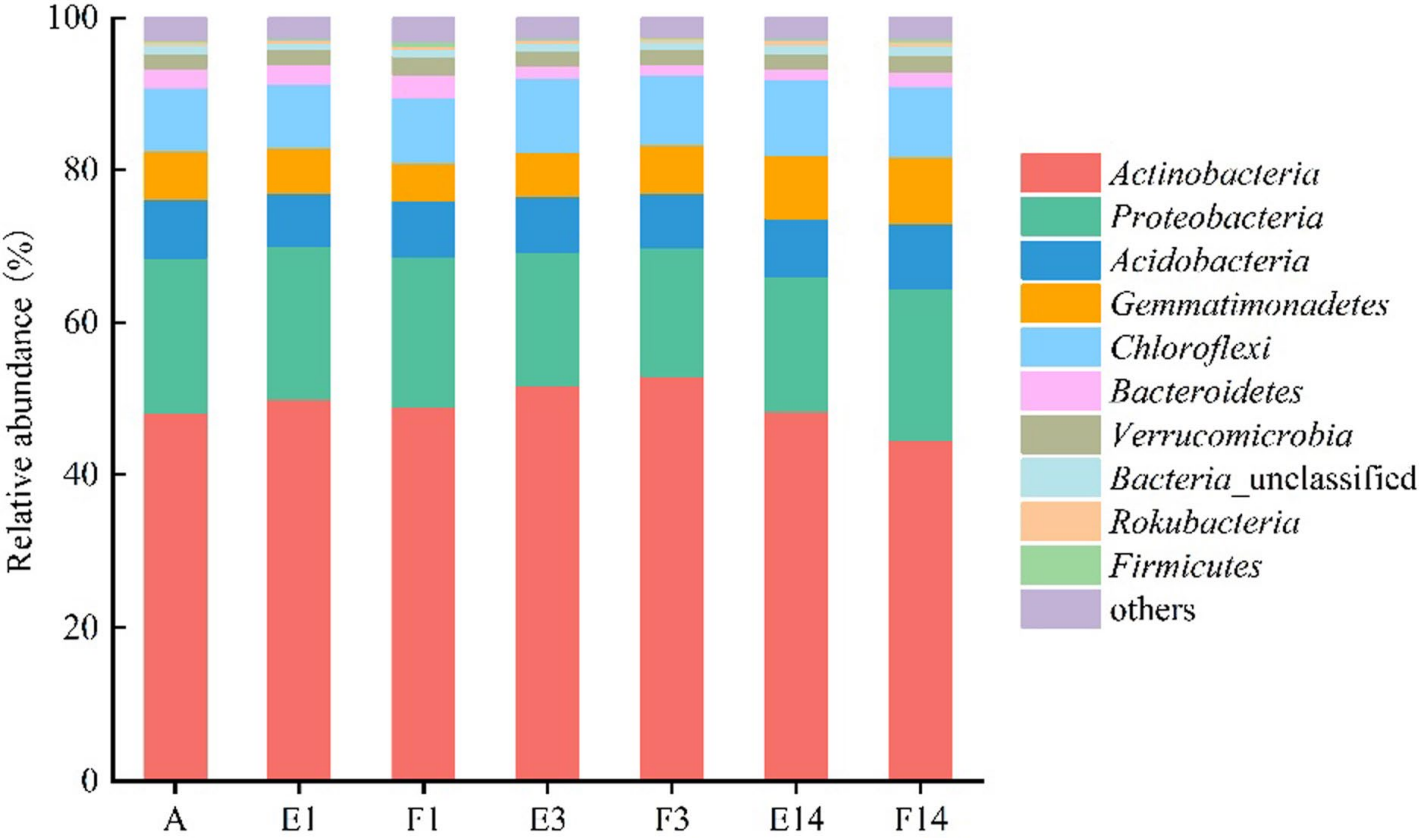
Relative abundances of soil bacterial communities at the phylum level.

Figure 4 shows the relative abundances of soil bacteria at the phylum level in different treatment groups. The dominant bacterial phyla in each treatment group were *Actinobacteriota*, *Proteobacteria*, *Acidobacteriota*, *Gemmatimonadota*, *Chloroflexi*, and *Bacteroidetes*, with their relative abundances ranging from 37.89 to 55.26%, 15.45 to 24.57%, 6.35 to 10.26%, 5.17 to 8.54%, 8.09 to 10.58%, and 1.24 to 2.72%, respectively.

At the phylum level, the changes in the dominant bacterial phyla in the soil of Group E were not significant when compared with the control Group A after the application of atrazine during the early stage of cultivation. Over time, the relative abundances of *Proteobacteria* and *Bacteroidetes* decreased, while that of *Chloroflexi* increased. This suggests that atrazine inhibited the growth of *Proteobacteria* and *Bacteroidetes* in soil, while promoting the growth of *Chloroflexi*. Compared with the control group, the abundance of *Actinobacteria* showed an initial increase followed by a decrease after adding strain G1-N1A. The abundance of *Proteobacteria* did not differ significantly from that in the control group during the early stage of cultivation and decreased over time, whereas the abundance of *Chloroflexi* increased. *Gemmatimonadota* play a specific role in the environment and are resistant to environmental stress. Following the addition of strain G1-N1A, the abundance of *Gemmatimonadota* decreased in the initial stage, remaining lower in the atrazine-treated group. This suggests that strain G1-N1A alleviated environmental stress to some extent in the short term and improved the soil’s ecological environment. In summary, atrazine may impact soil microorganisms to varying degrees under different treatment conditions. Adding strain G1-N1A affects the relative abundances of certain bacteria in the soil, stabilizing the soil’s microbial community.

**Figure 5:**
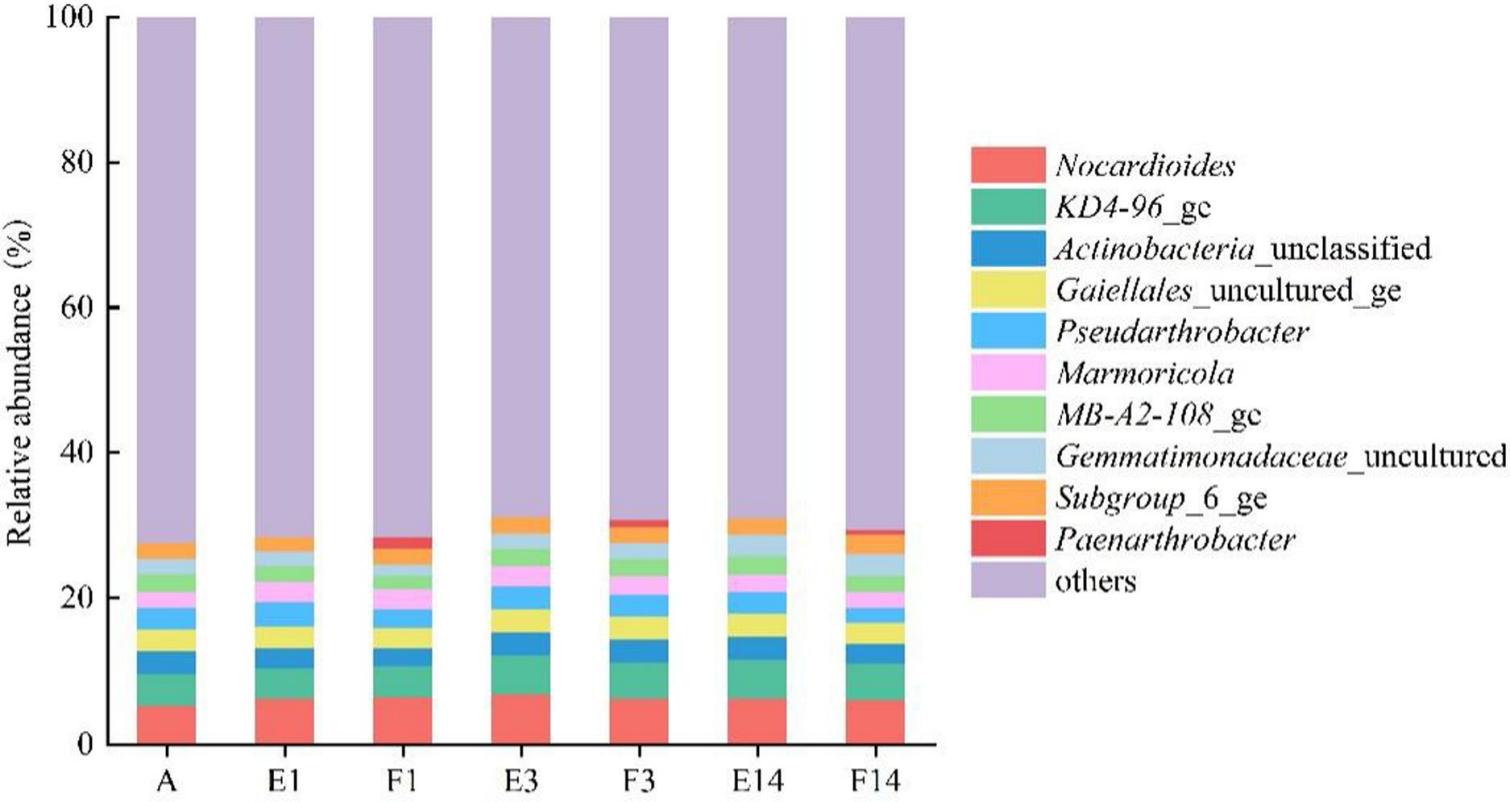
Relative abundances of soil bacterial communities at the genus level.

Figure 5 shows the relative abundances of representative soil bacteria at the genus level in different treatment groups. Following the addition of atrazine, the abundance of *Pseudarthrobacter* increased and then decreased slightly during the later stage of cultivation, although it remained higher than in the bacteria-supplemented treatment. This indicates that the addition of atrazine increased the abundance of *Pseudarthrobacter*. On the 3rd day, compared with other culture times, the changes in the relative abundances of the *Nocardioides* and *KD-96_ge* after the addition of atrazine were slightly greater than in the control group. Compared with the control group, adding strain G1-N1A increased the relative abundance of the *Paenarthrobacter* community gradually over time. Furthermore, the addition of strain G1-N1A resulted in *MB-A2-108_ge* being less abundant than in the drug-treated and control groups. In conclusion, both the addition of atrazine and of strain G1-N1A impacted the relative abundances of bacterial genera in the soil bacterial community.

#### 3.4.2 Analysis of *α*-diversity levels in soil bacterial communities

In the presence of atrazine, the Chao1 index initially decreased and then increased. Compared with the control group at the beginning of the culture process, the species richness in the E1 group decreased slightly, but this difference was not significant. Following the inoculation of strain G1-N1A, the Chao1 index was higher in the control than that in the E1 group. The presence of strain G1-N1A increased species richness (Table 1). After the application of atrazine, with an increase in action time, the OTU and Chao1 indices of the E3 group decreased, falling below those of the control group. At this time, the Chao1 index of the F3 group was higher than that of the control and E3 groups. With the increase in culture time, the atrazine content in the soil decreased, and the Chao1 index of the E14 treatment group with added atrazine began to increase. However, the Chao1 index of the F14 G1-N1A-inoculated treatment group did not increase significantly. The change trend of the OTU index was consistent with that of the Chao1 index. Adding the G1-N1A strain reduced the effect of atrazine on soil microorganisms during the early stage of culture, increasing the number of OTUs and the Chao1 index. The Shannon index decreased and then increased after atrazine was applied. On the 3rd day, the Shannon index values of Groups E and F differed significantly from those on Days 1 and 14, indicating various levels of microbial diversity in the soil at these times. At 14 days, the Shannon index of Group E was 7.76, whereas that of Group F was slightly lower at 7.74. This suggests that a decrease in G1-N1A would impact the recovery of soil microbial community richness and diversity. The higher a Coverage value, the higher the probability that a sequence will be detected in the sample. Coverage was found to be between 94.44 and 95.81% in all the treatments, indicating that the sequencing results sufficient sequencing depth the majority of bacterial taxa of the tested samples. In conclusion, the addition of strain G1-N1A promoted the OTU and Chao1 indices in all the soils where atrazine was applied, indicating that it was beneficial for improving the richness of microbial communities.

**Table 1:** Total OTUs and *α*-diversity indices of bacterial communities.

Sample	OTUs	Chao1	Shannon	Coverage
A	9,818 ± 284 a	11,463 ± 274 a	7.76 ± 0.02 a	0.9555
E1	9,358 ± 336 a	10,781 ± 251 a	7.63 ± 0.04 ab	0.9581
F1	9,552 ± 230 a	12,381 ± 2,236 a	7.60 ± 0.04 b	0.9474
E3	9,265 ± 556 a	10,822 ± 61 a	7.60 ± 0.08 b	0.9562
F3	9,480 ± 62 a	14,440 ± 5,889 a	7.59 ± 0.11 b	0.9444
E14	9,565 ± 596 a	11,410 ± 349 a	7.76 ± 0.08 a	0.9565
F14	9,675 ± 156 a	14,394 ± 4,944 a	7.74 ± 0.11 ab	0.9451

Data in the table are mean ± SE. Different lowercase letters in the same column indicate significant differences as assessed by Duncan’s new multiple range test (*p* < 0.05).

#### 3.4.3 PCoA of soil bacterial community structure in different treatments and time points

**Figure 6:**
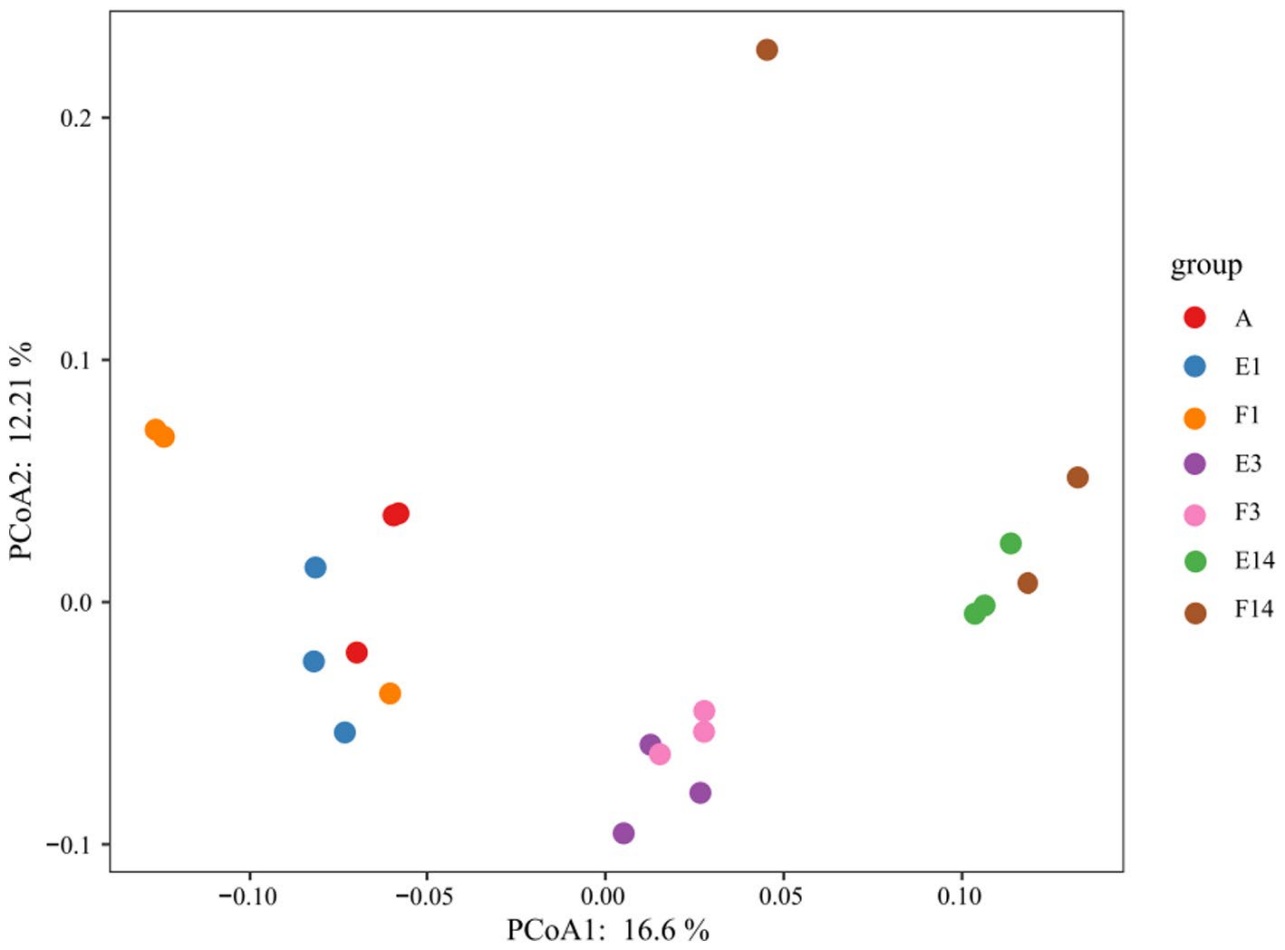
Principal coordinate analysis of the overall bacterial community composition. Each dot represents a sample, and samples of the same color indicate the same treatment.

The PCoA results showed that cultivation time was the core factor driving the differentiation of the soil bacterial community structure. Samples from 1 d, 3 d, and 14 d formed completely independent clusters along the PCoA1 axis, reflecting the phased succession characteristics of soil microorganisms under atrazine stress. On day 1 of the initial cultivation stage, the community structures of the blank control, the contaminated, and the remediation groups were highly similar, indicating that atrazine stress and strain inoculation had not yet exerted a significant impact on the initial community. After 3 days of cultivation, the community structures of the contaminated and remediation groups changed significantly and were completely separated from those of the 1 d groups. This suggested that stress had become the dominant factor driving community structure changes, but the strain had not yet produced a significant independent regulatory effect. By day 14 of cultivation, the samples were completely separated from the initial samples and the blank control group, indicating that atrazine stress was the core factor driving the significant alteration in the soil microbial community structure. Additionally, the contaminated and remediation groups’ samples at the same time points highly clustered together, revealing that in non-sterilized soil, the influence of inoculating G1-N1A on the overall microbial community structure was far less than that of atrazine stress and cultivation time. The strain mainly participated in atrazine degradation through synergistic interactions with indigenous microorganisms, without causing disruptive changes to the overall community structure (Figure 6). We further conducted ANOSIM significance test using the weighted UniFrac distance matrix. The results showed that R = 0.639, *p* = 0.001, which demonstrated significant differences in soil bacterial community structure among different treatments and incubation times. This result was consistent with the grouping pattern observed in the PCoA plot. Coverage values of all samples ranged from 94.44 to 95.81%, suggesting that the sequencing depth was sufficient to capture the majority of bacterial taxa in the soil samples.

### 3.5 Effects of strain G1-N1A-enhanced soil remediation on common bean growth

**Figure 7:**
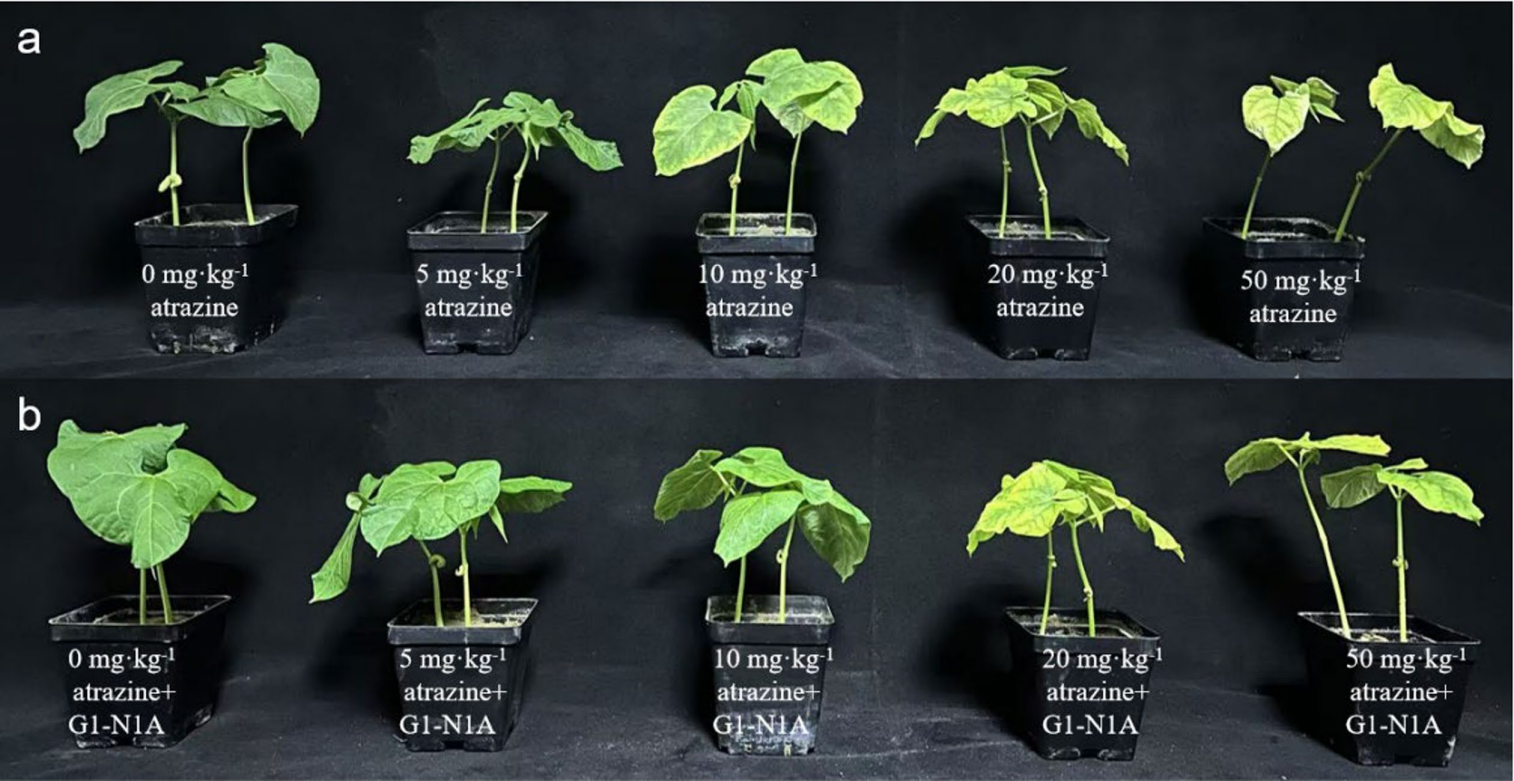
Effects of treatments on the growth of common bean. **(a)** Effects of different atrazine concentration gradients. **(b)** Effects of adding strain G1-N1A to soil containing different atrazine concentrations.

**Figure 8:**
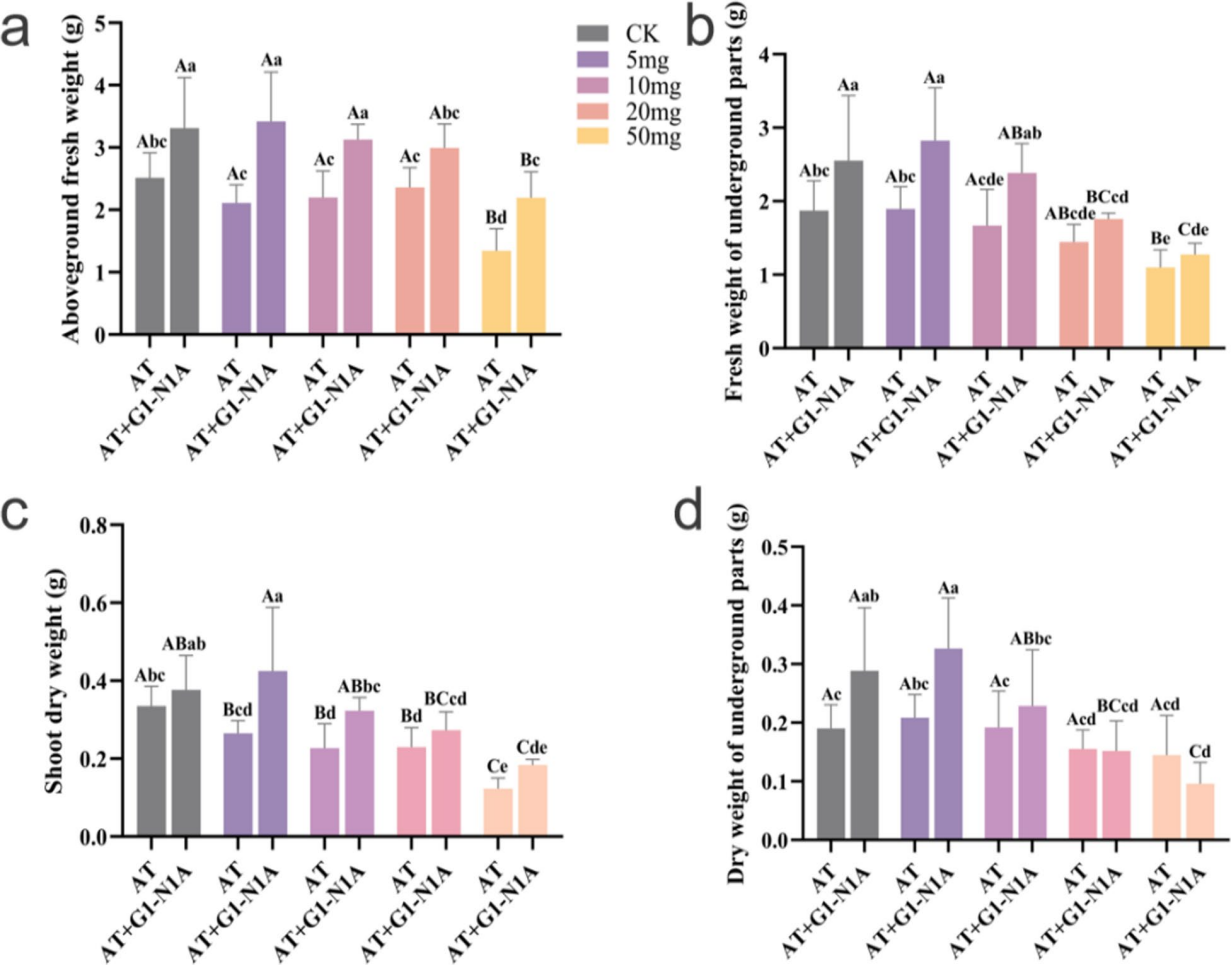
Effects of G1-N1A addition on the fresh and dry weights of common bean grown in soil containing different atrazine concentrations. **(a)** Aboveground fresh weight; **(b)** Underground fresh weight; **(c)** Aboveground dry weight; and **(d)** Underground dry weight. The lowercase and uppercase letters indicate significant difference between the same treatments at same and different concentrations, respectively (*p* < 0.05) as assessed by Duncan’s new multiple range test.

Figures 7, 8 shows the effects on plant aboveground and underground fresh weights, as well as aboveground and underground dry weights, of the degrading bacterium G1-N1A when plants are growing in soil containing different atrazine concentrations. In the treatment group with only the atrazine application, as the atrazine concentration increased, the aboveground fresh, underground fresh, aboveground dry, and underground dry weights all showed a gradient downward trend (Figures 8a–d). At the same atrazine concentration, the simultaneous application of G1-N1A, compared with no application, showed increases in the above four biomass indicators, except that the root dry weight of plants grown under a high concentration of atrazine + G1-N1A was lower than or equal to that of plants grown only under the comparable atrazine level. Under 50 mg·kg^−1^ atrazine stress, the atrazine + G1-N1A treatment still effectively improved the biomass accumulation level of plants. This indicates that the degrading bacterium G1-N1A significantly alleviated the inhibitory effects of atrazine on the biomass accumulations of the aboveground and underground plant parts, thereby enhancing the adaptability of plants to the atrazine-contaminated environment (Figure 8).

Two-way ANOVA was applied to evaluate the effects of atrazine concentration and G1-N1A inoculation on the shoot fresh weight of common bean. The overall fitted model was highly significant (*F*_9,50_ = 10.869), (*p* < 0.001), (*R*^2^ = 0.662), which indicated that the combination of atrazine contamination gradient and strain inoculation together explained 66.2% of the total variation in plant fresh weight. The six combined treatments integrating different atrazine concentrations and inoculation statuses showed extremely significant differences in shoot fresh weight (*F*_5,50_ = 11.155), (*p* < 0.001). The interaction effect of inoculation treatment with strain G1-N1A on the fresh root weight of common beans can be observed. The overall regression model was highly significant (*F*_9,50_ = 8.929), (*p* < 0.001), (*R*^2^ = 0.616), demonstrating that the two experimental factors collectively explained 61.6% of the total variation in root fresh weight. The six combined treatments consisting of different atrazine contamination levels and inoculation status exhibited extremely significant differences in root fresh weight (*F*_5,50_ = 5.594), (*p* < 0.001).

Two-way ANOVA was applied to evaluate the combined effects of atrazine concentration and G1-N1A inoculation on the total dry weight of common bean seedlings. The overall fitted model was highly significant (*F*_9,50_ = 10.151), (*p* < 0.001), (*R*^2^ = 0.646), which indicated that atrazine pollution gradient and strain inoculation together explained 64.6% of the total variation in plant dry weight. The six combined treatments formed by different atrazine concentrations and inoculation status showed significant differences in seedling dry weight (*F*_5,50_ = 5.161), (*p* = 0.001). Atrazine concentration and G1-N1A inoculation on root dry weight of common bean seedlings. The overall model was highly significant (*F*_9,50_ = 6.159), (*p* < 0.001), (*R*^2^ = 0.526), which indicated that atrazine pollution gradient and strain inoculation collectively explained 52.6% of the total variation in root dry weight. The six combined treatments integrating different atrazine concentrations and inoculation statuses showed significant differences in root dry weight (*F*_5,50_ = 3.566), (*p* = 0.008).

### 3.6 Effects of strain G1-N1A-enhanced remediation on the SOD, POD, CAT, and APX activities, MDA content, and chlorophyll level in common bean leaves

**Figure 9:**
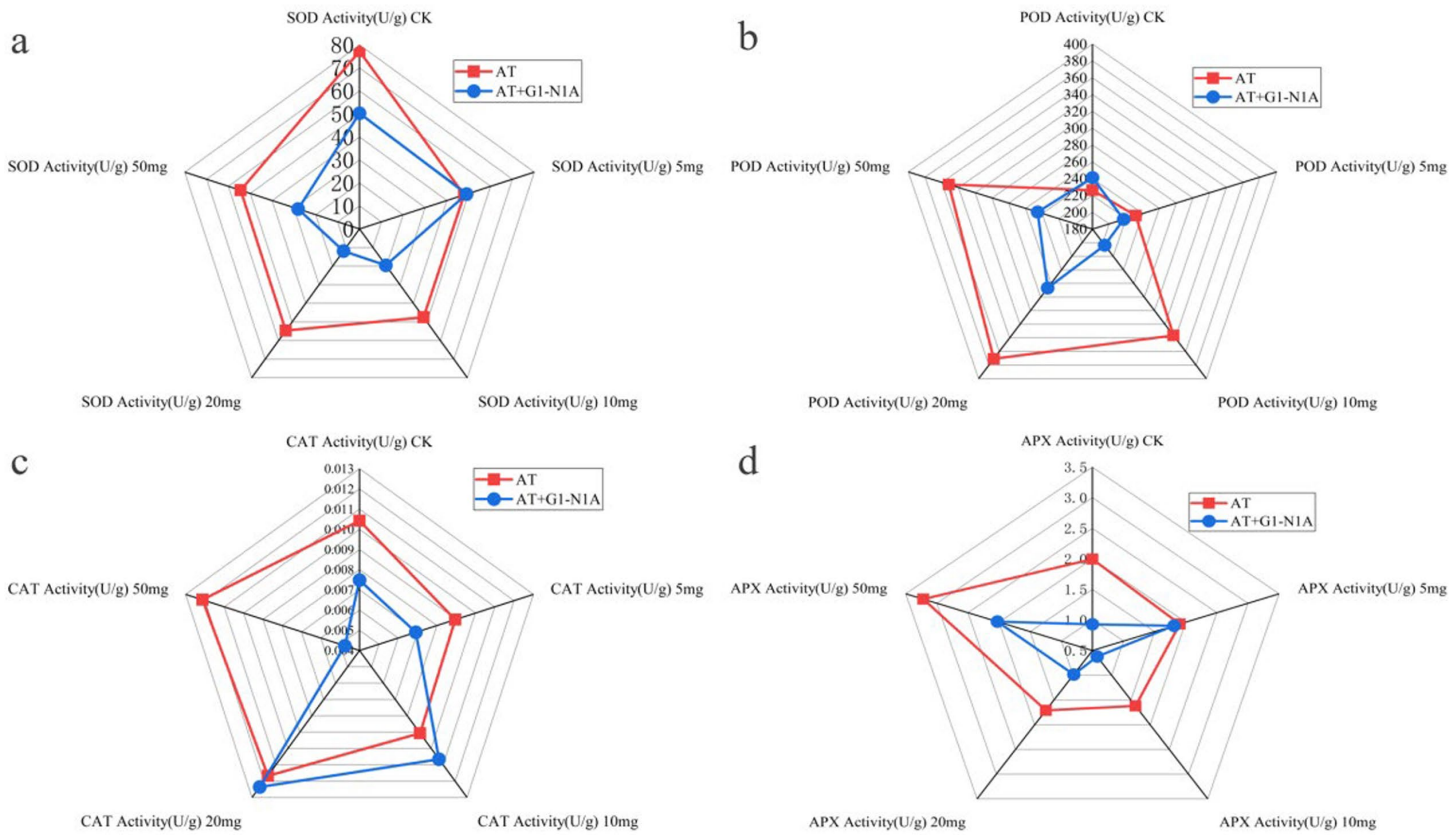
Effects of G1-N1A addition on enzyme activities in common bean leaves from plants grown in soil containing different atrazine concentrations. **(a)** SOD; **(b)** POD; **(c)** CAT; and **(d)** APX activity levels.

A change in the activity level of SOD, a key enzyme in the plant antioxidant system that scavenges superoxide anion radicals, can directly reflect the intensity of oxidative stress. In the control treatment without atrazine stress, there was no significant difference in SOD activity between CK and CK + G1-N1A. With an increase in the atrazine concentration, the SOD activity showed an obvious upward trend. The activities at other stress concentrations were significantly higher than those in the atrazine + G1-N1A group, except for those of the 5 mg·kg^−1^ atrazine and 5 mg·kg^−1^ atrazine + G1-N1A groups, which were similar. This result indicates that atrazine stress induced the massive accumulation of superoxide anion radicals in plants, which in turn activated the expression and activity of SOD to maintain cellular redox homeostasis. However, the application of G1-N1A effectively reduced the stress effect of atrazine at high concentrations and decreased the production level of reactive oxygen species. Therefore, compared with the atrazine group, in the G1-N1A-treated group, the SOD activity showed a significant decreasing trend as the atrazine concentration increased (Figure 9a).

The POD activity in the control atrazine and atrazine + G1-N1A groups was similar. As the atrazine concentration increased to 5, 10, 20, and 50 mg·kg^−1^, the POD activity in the atrazine group increased significantly, and the activity at each concentration was higher than that in the corresponding atrazine + G1-N1A group. This suggests that under atrazine stress, the accumulation of peroxides, such as hydrogen peroxide, in plants increased, thereby inducing the upregulation of POD activity to enhance antioxidant defense capabilities. However, the degradation of atrazine by G1-N1A reduced the stress intensity and the peroxide accumulation level; consequently, the POD activity showed a significant downward trend compared with in the atrazine group (Figure 9b).

Under control conditions, the basal CAT activity of the atrazine treatment group was slightly higher than that of the atrazine + G1-N1A group. When the atrazine concentration increased to 5 mg·kg^−1^, the CAT activity of the atrazine group significantly decreased to the lowest level, whereas the atrazine + G1-N1A group showed a similar enzyme activity level. As the concentration of atrazine further increased, the CAT activity in the atrazine group continued to rise and reached its highest value at a 50 mg·kg^−1^ atrazine concentration. The CAT activity of the atrazine + G1-N1A group at 10 and 20 mg·kg^−1^ atrazine concentrations was significantly higher than that of the corresponding atrazine group. Under the high 50 mg·kg^−1^ atrazine concentration, the CAT activity of the atrazine group reached the highest level. At this time, the CAT activity of the atrazine + G1-N1A group was significantly lower compared to the high expression levels at the 10 and 20 mg·kg^−1^ atrazine concentrations (Figure 9c).

In the control group, the APX activity of the atrazine alone treatment group was significantly higher than that of the atrazine + G1-N1A group. With an increase in atrazine concentration, the APX activity of the atrazine alone treatment group showed a decreasing trend at the 5 to 20 mg·kg^−1^ concentration before rebounding at 50 mg·kg^−1^. The APX activity of the atrazine + G1-N1A treatment group was lower than that of the corresponding atrazine alone treatment group under all atrazine concentrations, with the activities of the two groups being equal only at 5 mg·kg^−1^. This indicates that G1-N1A reduced the actual stress intensity of atrazine through degradation, thereby alleviating the oxidative stress caused by atrazine, and maintained the activity of APX, a key enzyme in antioxidant defense, at a relatively low level compared with the atrazine control (Figure 9d).

**Figure 10:**
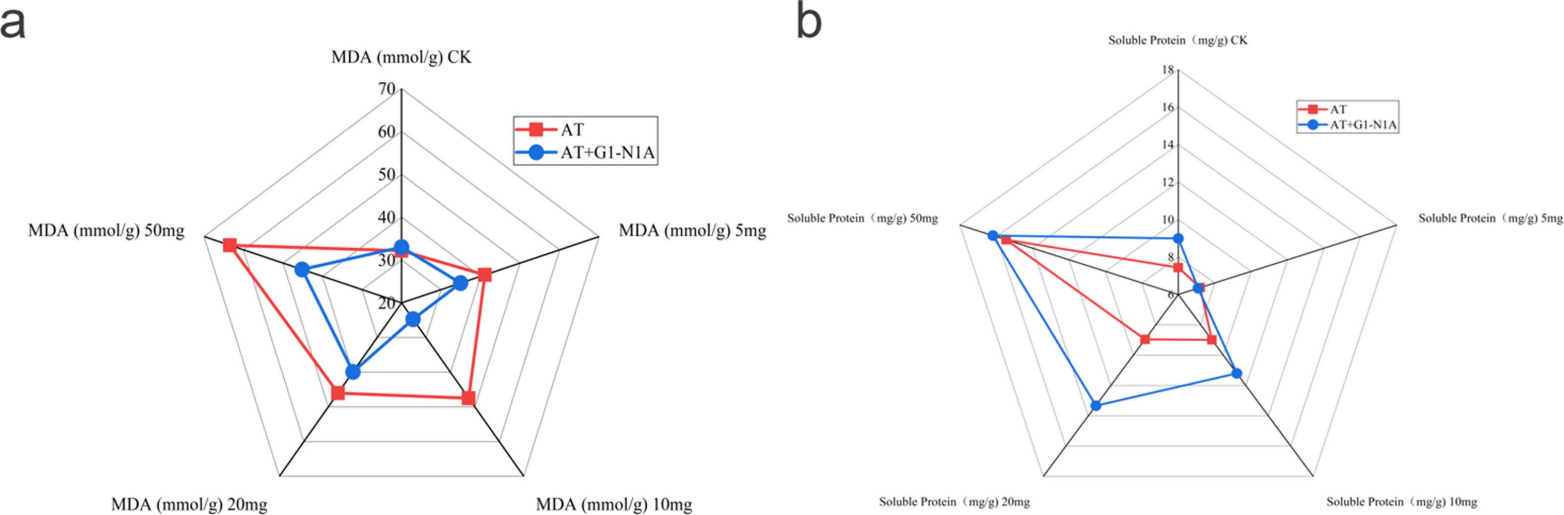
The effects of adding strain G1-N1A on MDA **(a)** and soluble protein levels **(b)** in common bean leaves of plants grown in soil containing under different atrazine concentrations.

Under control conditions, the MDA content in the atrazine alone treatment group was significantly higher than that in the atrazine + G1-N1A treatment group. At atrazine stress concentrations ranging from 5 to 50 mg·kg^−1^, the MDA content in the atrazine treatment gradually decreased as the stress concentration increased. The MDA content in the atrazine + G1-N1A treatment was slightly higher than that in the atrazine treatment only at 50 mg·kg^−1^, and it was lower than that of the atrazine treatment group, to varying degrees, at other stress concentrations (Figure 10a).

Under control conditions, the soluble protein content in the leaves of common bean treated with atrazine + G1-N1A was higher than that in the atrazine alone treatment group. Under 5, 10, and 20 mg·kg^−1^ atrazine, the soluble protein content in the atrazine alone treatment group remained at a relatively low level overall, whereas the soluble protein content in the atrazine + G1-N1A treatment group at 10 and 20 mg·kg^−1^ atrazine concentrations was higher than in the corresponding atrazine alone single treatment group. The difference was particularly significant at the 20 mg·kg^−1^ atrazine concentration, indicating that G1-N1A effectively alleviated the inhibitory effects of atrazine on the accumulation of soluble protein in common bean leaves (Figure 10b).

**Figure 11:**
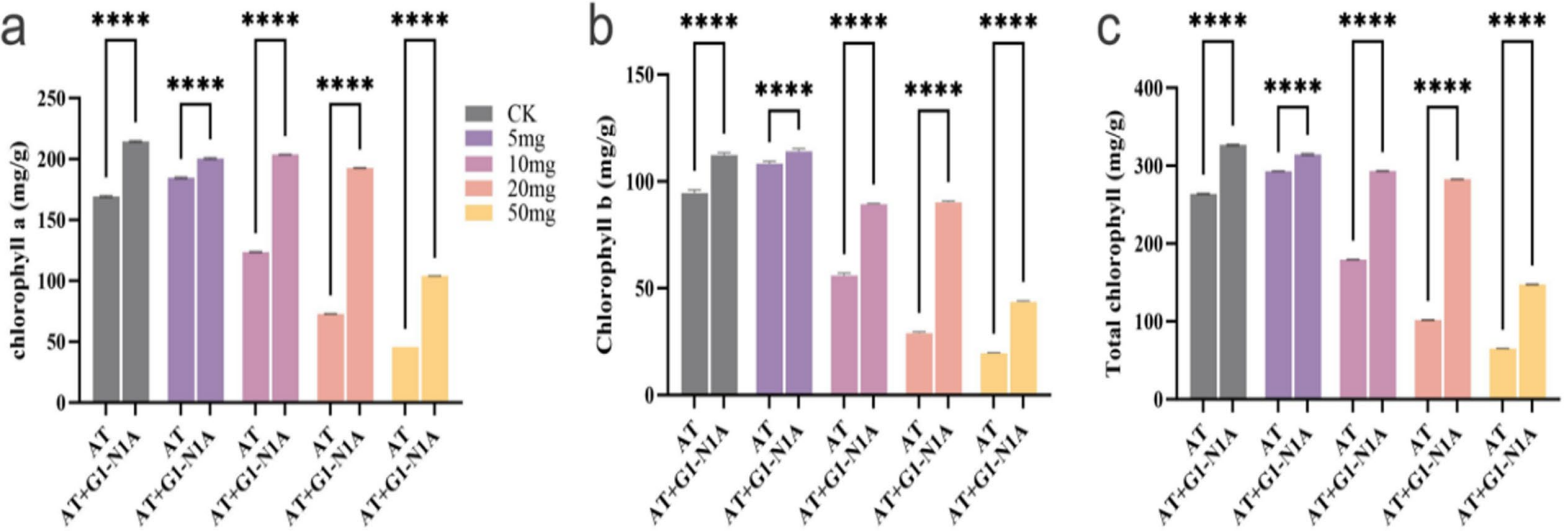
The effects of adding strain G1-N1A on chlorophyll in common bean leaves of plants grown in soil containing different atrazine concentrations. **(a)** Chlorophyll a; **(b)** Chlorophyll b; and **(c)** Total chlorophyll. The symbol “****” indicates an extremely significant difference between the G1-N1A treatment group and the corresponding atrazine only treatment group as determined by a one-way analysis of variance (*p* < 0.0001).

Figure 11 shows changes in chlorophyll a, chlorophyll b, and total chlorophyll contents in leaf samples from plants grown at different atrazine concentrations (0, 5, 10, 20, and 50 mg·kg^−1^) and treated with G1-N1A. In the atrazine only treatment group, as the atrazine concentration increased from 0 to 50 mg·kg^−1^, the chlorophyll a, chlorophyll b, and total chlorophyll contents each showed an extremely significant downward trend, indicating that atrazine stress has a strong inhibitory effect on the synthesis or stability of photosynthetic pigments in common bean leaves. Under the same atrazine concentrations, the addition of G1-N1A resulted in the chlorophyll a, chlorophyll b, and total chlorophyll contents being extremely significantly higher than those in the corresponding atrazine only treatment group. This indicates that G1-N1A effectively alleviated the damage caused by atrazine stress to the chlorophyll system, thereby maintaining relatively high levels of photosynthetic pigments (Figures 11a–c).

## 4 Discussion

Compared with previously reported atrazine-degrading strains, especially those in the *Paenarthrobacter* and *Arthrobacter* genera, strain G1-N1A shows obvious superiorities. Atrazine has a relatively long retention time in soil, with a half-life degradation period of between 13 and 261 days. To investigate the remediation effects of strain G1-N1A on atrazine-contaminated soil, this study simulated an agricultural environment having contaminated soil. Strain G1-N1A showed a superior rapid degradation capacity in soil, which was supported by its significantly higher short-term removal efficiency relative to previously reported atrazine-degrading strains. This rapid degradation can be attributed to the functional *trzN*, *atzB*, and *atzC* gene cassette detected in this study, which enabled the efficient hydrolytic transformation of atrazine. *Paenarthrobacter* sp. AT-5 removes 50 mg·kg^−1^ of atrazine from soil within 7 days, achieving a removal rate of 95.90% (Jia et al., 2021). *Arthrobacter* sp. HB-5 takes 56 days to fully degrade 10 mg·kg^−1^ of atrazine (Wang et al., 2011). *Pseudomonas* sp. ADP needs 7 days to degrade 7 mg·kg^−1^ of atrazine in soil (Lima et al., 2009). Compared with these strains, especially at the same atrazine concentration, G1-N1A exhibits an excellent bioremediation ability. In addition, most existing studies only focus on degradation efficiency, while G1-N1A simultaneously improves soil enzyme activities, regulates microbial community structure, and protects crop growth, which is critical for safe production in corn–bean rotation systems.

Soil enzymes are widely used as important indicators of soil quality and soil biological activity (Guangming et al., 2017). Most soil enzymes are secreted by soil microorganisms (Yang R. et al., 2021), and the application of atrazine affects the relative abundances and structures of soil microbial communities (Liu et al., 2020). The three soil enzymes studied in this paper exhibited different responses to atrazine. The measured enzymatic data indicated that atrazine stress suppressed key soil enzyme functions, whereas bioaugmentation with G1-N1A alleviated this inhibition. This effect was linked to the reduced toxicant pressure and enhanced abundance of enzyme-producing microbial taxa, rather than a direct stimulation by the strain itself. The recovered enzyme activities reflected an improved soil nutrient cycling capacity, which was consistent with the observed enhanced crop growth.

Our analysis of microbial community composition revealed significant changes after the application of atrazine, consistent with previous reports (Kolekar et al., 2019). Atrazine caused significant changes to the bacterial community that were attributed to the combined effects of different atrazine concentrations and soil microorganisms (Díaz-López et al., 2019). Studies by Douglass et al. (2015) and Piutti et al. (2003) have shown that microorganisms can absorb atrazine as a nutrient source for growth. The increased degradation rate of atrazine may, therefore, be due to changes in the microbial community composition. Some rhizosphere microorganisms can use atrazine as a substrate, thereby increasing its degradation rate in the rhizosphere soil (Kolekar et al., 2019; Li et al., 2008). The observed shifts in bacterial community structure were driven by reduced atrazine toxicity following bioaugmentation. The increased relative abundance of *Paenarthrobacter* and *Nocardioides* suggested the enrichment of functional degraders, which supported sustained atrazine attenuation. The elevated *α*-diversity indices demonstrated that G1-N1A promoted community resilience rather than disrupting native microbiota, supporting the ecological safety of this bioaugmentation strategy.

At the genus level, the addition of strain G1-N1A increased the relative abundance of *Paenarthrobacter* compared with the control group, but this increased abundance decreased slowly over time. The strain also reduced the abundance of *MB-A2-108_ge*. By Day 3, the changes in the abundances of *Nocardioides* and *KD-96_ge* indicated that the addition of atrazine had a slightly greater impact on the relative abundances of the microbiota than in the control group at other incubation times. Additionally, sucrase and urease activities were higher during this incubation period than on Day 1, possibly because atrazine stimulated the microorganisms at this time, thereby enhancing their activities. Devers et al. (2007) demonstrated that *Nocardioides* possesses the *atzB*, *atzC*, and *trzN* genes, enabling it to grow using atrazine as a nutrient source. It is worth noting that microbial adaptation to atrazine stress can increase the abundance of degrading bacteria (Liu et al., 2020; Douglass et al., 2015). The atrazine degradation pathway of strain G1-N1A is mediated by the key genes *trzN*, *atzB*, and *atzC*. The *trzN* gene catalyzes the hydrolysis of atrazine to hydroxyatrazine; *atzB* further transforms hydroxyatrazine into N-isopropylammelide; and *atzC* finally converts it into cyanuric acid. In terms of microbial interactions, G1-N1A plays a may interact synergistically with indigenous soil microorganisms. By rapidly degrading atrazine and reducing ecotoxicity, G1-N1A creates a favorable niche for native atrazine-degrading genera, such as *Nocardioides* and *Paenarthrobacter*, which further metabolize cyanuric acid. This synergistic succession accelerates soil functional recovery.

In this study, the abundance of *Nocardioides* in E3 soil was higher than in CK soil. This suggests that these bacterial species possess enzymes capable of catalyzing the decomposition of atrazine into less toxic compounds. Segata et al. (2011) reported the isolation of the atrazine-degrading bacterium *Nocardioides* from soil and found that its atrazine degradation rate is strongly related to the number of atrazine applications (Ghirardelli et al., 2021; Zhao et al., 2017).

Treatment with atrazine slightly reduced the *α*-diversity of the bacterial community in the soil. The presence of strain G1-N1A increased the total number of OTUs and the Chao1 index, thereby promoting bacterial community richness in atrazine-contaminated soil. The Shannon index changed significantly on the 3rd day, indicating obvious changes in bacterial community diversity. The PCoA also showed that the addition of atrazine impacted the structure of the soil bacterial community. This may be because the bacteria in the microbial inoculum reduce the impacts of pollutants on soil microorganisms and modulated the soil microbial community structure (Subashchandrabose et al., 2013).

The efficient degradation ability of G1-N1A is the basis for recruiting beneficial microorganisms. Atrazine, as a toxic pollutant, inhibits the growth of most microorganisms. However, G1-N1A significantly reduces soil toxicity stress by degrading atrazine into low-toxic cyanuric acid. Microorganisms in the soil that have the ability to degrade cyanuric acid, such as strains of the genera *Nocardioides* (Ma et al., 2023) and *Paenarthrobacter* (Chen S. et al., 2021), can further degrade cyanuric acid. After inoculation with G1-N1A, the number of OTUs and the Chao1 and Shannon indices of the soil bacterial community in the treatment group were significantly higher than those in Group E, which only contained atrazine. Moreover, the abundances of dominant phyla such as *Actinobacteriota* and *Proteobacteria* tended to be stable. These phyla are the main producers of soil sucrase, urease, and cellulase. The activities of these three enzymes in Group F remained higher than those in Group E during the incubation period. This was especially true for urease, which was significantly activated from Days 3 to 14. This may be directly related to the enrichment of beneficial microorganisms such as *Paenarthrobacter* (Chen S. et al., 2021) and *Nocardioides* (Ma et al., 2023). The growth and metabolism of such microorganisms increase soil enzyme activities by secreting extracellular enzymes, thereby accelerating organic matter decomposition and nutrient cycling and increasing soil available nutrients (Velmourougane et al., 2019). Furthermore, through the chain reaction of microbial enrichment, enhanced enzyme activity, and nutrient activation, they provide key soil conditions for the accumulation of common bean biomass.

Under atrazine stress, the activities of antioxidant enzymes, such as SOD, POD, and CAT, increased, the MDA content increased, and the chlorophyll content decreased in common bean leaves. This reflects the crop’s response to oxidative stress (Nadarajah, 2020; Liu et al., 2026). The agronomic and physiological traits of common beans were improved, which may be attributed to the ability of G1-N1A to effectively alleviate phytotoxicity caused by atrazine in plants. The reduced activities of antioxidant enzymes and malondialdehyde (MDA) content indicate mitigated oxidative damage; meanwhile, the elevated chlorophyll content suggests that photosynthetic function is preserved. The improvement in plant performance may be associated with atrazine degradation and detoxification by strain G1-N1A. Since atrazine residues in pot soil were not quantified in this study, we cannot confirm whether the growth improvement solely resulted from reduced herbicide toxicity, nor can we rule out the direct growth-promoting effect of G1-N1A itself.

The G1-N1A strain exhibited multiple advantages in the remediation of atrazine-contaminated soil, including efficient degradation, protection of soil ecological functions, and alleviation of crop stress. It is particularly well-suited to managing atrazine residues in the corn–legume rotation system used in Northeast China, effectively solving the problem of phytotoxicity to subsequent crops. However, the research also has certain limitations. The regulatory mechanisms of the strain’s gene expression in degradation processes and its interactions with indigenous microorganisms have not been thoroughly investigated. In future, combining transcriptomics, metabolomics, and other technologies will be necessary to reveal the molecular mechanisms. The issues such as the large-scale cultivation of the strain, the development of formulation, and the costs of application still need to be resolved to realize the transformation from laboratory research to practical application. This study was conducted under controlled laboratory and pot conditions to ensure the accuracy and repeatability of degradation, soil ecology, and phytotoxicity alleviation data. These results provide a solid foundation for subsequent field plot experiments in actual agricultural environments to verify the strain’s stability and application efficiency. For practical agricultural applications, future research will focus on the large-scale fermentation of strain G1-N1A, development of stable formulations, and evaluation of field application costs and compatibility with conventional farming practices. It should be noted that the present study used freshly spiked atrazine rather than field-aged residues. Aging in real soil can reduce the bioavailability of atrazine, which may lead to lower degradation rates in the field than those observed under our controlled laboratory conditions.

Nevertheless, strain G1-N1A is a highly efficient functional strain, which provides crucial microbial resources for the green remediation of atrazine-contaminated soil. In-depth analysis of its remediation mechanism can lay a theoretical foundation for the bioremediation of similar pollutants. Follow-up research can further verify the bioaugmentation remediation mechanism of G1-N1A through multi-group optimization experiments. For instance, set up a viable cell control group with uncontaminated soil and a high-temperature inactivated G1-N1A control group to distinguish the degradation effect, microbial activity and growth-promoting effect of the strain; adopt the plate counting method, real-time quantitative PCR (qPCR) or specific marker gene technology to monitor the survival and colonization capacity of the strain; rely on microbial co-occurrence networks and *in vitro* co-culture assays to elaborate the synergistic interactions and metabolic cross-feeding mechanisms among strains; measure the residual atrazine content in potted soil to verify the soil detoxification efficiency of this strain. These measures can further confirm the bioaugmentation remediation efficacy and ecological safety of G1-N1A in atrazine-contaminated soil.

## 5 Conclusion

This study shows that *Paenarthrobacter nitroguajacolicus* G1-N1A can degrade 85.20% of 10 mg·kg^−1^ atrazine in soil within 1 day. Cyanuric acid was putatively identified as its major degradation product via UHPLC–MS. The strain enhanced activities of soil sucrase, urease and cellulase, altered the composition of soil bacterial communities, increased the abundance of Arthrobacter-related taxa, and facilitated community succession and diversity. G1-N1A can also alleviate the phytotoxicity of atrazine on common beans and increase plant biomass. It reduced the activities of SOD, POD, CAT and MDA content, and kept chlorophyll at a stable level. Overall, G1-N1A is a promising strain for the bioremediation of atrazine-contaminated soil. This work provides theoretical and technical support for pollution remediation and safe crop production in corn–bean rotation systems.

## Data Availability

The datasets presented in this study can be found in online repositories. The names of the repository/repositories and accession number(s) can be found at: https://zenodo.org/records/19631330, 10.5281/zenodo.19631329, https://www.ncbi.nlm.nih.gov/genbank/, OP107895.1 https://www.ncbi.nlm.nih.gov/genbank/, OR735533 https://www.ncbi.nlm.nih.gov/genbank/, OR735534 https://www.ncbi.nlm.nih.gov/genbank/, OR735535.

## Footnotes

1
https://www.genome.jp/kegg/pathway.html
